# Spatial distribution of *Gasterophilus pecorum* (Diptera) eggs in the desert steppe of the Kalamaili Nature Reserve (Xinjiang, China)

**DOI:** 10.1186/s12862-021-01897-4

**Published:** 2021-09-06

**Authors:** Heqing Huang, Ke Zhang, Changliang Shao, Chen Wang, Make Ente, Zhenbiao Wang, Dong Zhang, Kai Li

**Affiliations:** 1 Key Laboratory of Non-Invasive Research Technology for Endangered Species, School of Ecology and Nature Conservation, Beijing Forestry University, Beijing, 100083 China; 2 Chongqing Academy of Environmental Science, Chongqing, 401147 China; 3 Mt. Kalamaili Ungulate Nature Reserve, Changji, 381100 Xinjiang China; 4 Xinjiang Research Centre for Breeding Przewalski’s Horse, Ürümqi, 831700 Xinjiang China

**Keywords:** *Gasterophilus pecorum*, Egg, Spatial distribution, Geostatistics, Water resource

## Abstract

**Background:**

The dominant *Gasterophilus* species in the desert steppe (Xinjiang, China) *Gasterophilus pecorum* poses a serious threat to the reintroduced Przewalski’s horses. We investigated the distribution pattern of *G. pecorum* eggs in June 2017.

**Methods:**

Two sampling methods, transect and grid, were used, and the results were analyzed via geostatistics by semivariance. The nest quadrat was used to determine the optimal quadrat size.

**Results:**

Eggs were found in 99 quadrats (63.1%) and 187 clusters (1.5%) of *Stipa caucasica* on the steppe. The mean oviposition count of a cluster was 3.8 ± 1.6. Three-eggs is the mode of which females oviposit on each ovigerous *S. caucasica* (22.0%)*.* Semivariogram analysis revealed that the distance of spatial dependence for eggs was 921 m, 1233 m and 1097 m for transect 1, transect 2 and grid methods, respectively, while spatial continuity was 62%, 77% and 57.0% for transect 1, transect 2 and grid, respectively. The eggs showed a patchy, aggregated distribution pattern. This suggested the spherical model is most applicable. The proportion of ovigerous *S. caucasica* was significantly correlated with the distance from water resources (*r* = − 0.382, *p* = 0).

**Conclusion:**

Our findings indicated that diversification of *G. pecorum* oviposition was a new adaptative strategy for its survival in the desert steppe ecological niche. This made it more efficient at infecting hosts in the local environment. Areas surrounding water resources, especially around the drinking paths of equids (500 m radius surrounding the water), were concentrated epidemic areas. It is suggested that more attention to be paid to the ecological characteristics of *G. pecorum* in order to develop control measures that would reduce the infection risk for Przewalski’s horses.

**Supplementary Information:**

The online version contains supplementary material available at 10.1186/s12862-021-01897-4.

## Background

*Gasterophilus* larvae are internal parasites that infect the gastrointestinal tract of equids. Six of nine *Gasterophilus* species, *G. haemorrhoidalis*, *G. inermis*, *G. intestinalis*, *G. nasalis*, *G. nigricornis*, and *G. pecorum*, are known to be present in China [1, 2]. All these species have been reported to infect wild populations of Przewalski’s horse (*Equus przewalskii*) in the Kalamaili Nature Reserve (KNR) [3]. Previous studies have found *G. pecorum* to be the dominant species in the KNR. High prevalence and intensity of *G. pecorum* have seriously limited the restoration of Przewalski’s horse populations [4].

*Gasterophilus pecorum* is mainly distributed in Central Asia and South Africa [5–7]. As the only *Gasterophilus* species that lays eggs on grass (other species lay eggs on hosts), the life-history of *G. pecorum* has been described as “unusual” [8, 9]. Biological and ecological data related to *G. pecorum* are largely lacking and restricted only to early research conducted in Kazakhstan [10]. Previous studies have reported that the main epidemic areas of *G. pecorum* in the KNR were those in the proximity of water resources [11]. The adults usually oviposit on *Stipa caucasica* within 2000 m of the water resource [3].

Understanding the spatial distribution of insects enables the estimation of their population densities and facilitates decision making with regard to population control [12]. Since the 1980s, geostatistics has been widely used in the field of insect ecology as an important analytical method for their spatial distribution [13, 14]. In geostatistics, semivariance has been used to analyze the spatial autocorrelation of insects due to environmental factors [15, 16]. This study is the first to analyze the spatial distribution of *G. pecorum* oviposition around the water resources of KNR using a geostatistical methodology. The results of this study may enrich the ecological data of *G. pecorum* and help formulate control measures.

## Methods

### The study site

The KNR (latitude: 44° 36′ to 46° 00′ N, longitude: 88° 30′ to 90° 03′ E, altitude: 600–1464 m) covers an area of approximately 17,000 km^2^. The vegetation coverage in the KNR is 20–30%, and mostly consists of shrubs (*Anabasis brevifolia*, *Ceratoides laten*) and herbage (*Stipa caucasica*) [17]. The area is characterized by a temperate continental arid climate, with a mean annual precipitation of 159 mm and a mean annual evaporation of 2090 mm [18].

In the KNR, there is a shortage of water resources, with only three permanent reservoirs. The Hongliu (HL, a water resource) water, which is frequently used by equids, is the most important water resource in the area. Therefore, the equines often gather in the water source area, and vegetation around the water source area within 100 m meters is very scarce after being tramped and covered in feces. The area south of the HL water occupied by heaps of dirt, with sparse vegetation. After drinking, the equids mainly prefer the area to the north of the HL for foraging and resting [11].

### Quadrat size

To ensure the reliability of the research data, we performed a preliminary experiment to determine the optimal quadrat size. According to previous studies based in the KNR, peak adult emergence of *G. pecorum* occurs at the beginning of early June [19, 20]. First, we randomly selected 10 sites, with > 100 m intervals between every pair, within 1000 m of the HL water in June 2017. Secondly, the nest quadrat was used to investigate the density of *G. pecorum* eggs in *S. caucasica*. The quadrat sizes were 1 m × 1 m, 2 m × 2 m, 3 m × 3 m, 4 m × 4 m, and 5 m × 5 m [21]. Quadrats of different sizes in each sampling site were sampled once. We visually inspected all clusters of *S. caucasica* in each quadrat in situ to confirm if they contained eggs, and recorded the vegetation and oviposition information.

The frequency of eggs (F), presence of ovigerous *S. caucasica* ($\overline{x }$), mean number of ovigerous *S. caucasica* (μ), standard deviation (S) and coefficient of variance (CV) were used to select the optimal quadrat size. These indices were calculated as follows:

$${\text{F}} = {\text{ovigerous}}\;{\text{quadrats}}/{\text{all quadrats}} \times {1}00\% ;$$

$$\overline{x }=\mathrm{ovigerous} \, S. caucasica/\mathrm{all} \, S. caucasica \times 100\mathrm{\%};$$

$$\upmu = {\text{ovigerous }}S. caucasica/{\text{all quadrats}};$$

$${\text{CV}} = {\text{S}}/\upmu$$

### Data collection

Two sampling methods were used to investigate the oviposition activity of *G. pecorum* in the HL water from June to July 2017.

#### Transect

**Fig. 1:**
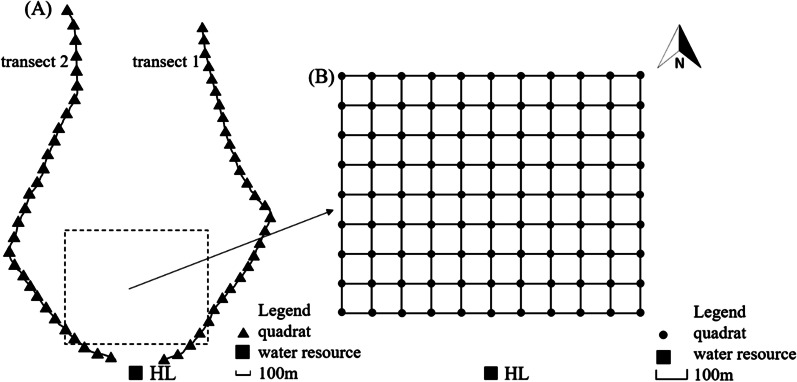
The transect method (**A**) and the grid layout (**B**) for sampling

There are two drinking paths of equids near the HL water site that is frequently used by wildlife in the reserve, namely are the two transects we investigated (Additional file 2). The Przewalski’s horses migrate along the paths almost every day, therefore, the investigation of these two transects could reflect the egg distribution and density of Gasterophilus pecorum eggs in part of a highly active area of the Przewalski’s horses in KNR. Due to the behavioral characteristics of equines, the vegetations within 100 m buffer around the water source were hard for growing. In order to reduce the error, we focus on the vegetation over 200 m distance from the water source site and set a quadrat (4 m × 4 m) in every 100 m along the two paths. In total, 58 sample points were located in these 2 transects from the HL water to the north (Fig. 1A).

#### Grid

A grid (length: 1000 m, width: 800 m) of 99 sample points (4 m × 4 m, at 100 m intervals) were located in the northern area of the HL water (Fig. 1B).

The center of water source site was set as the origin of the rectangular coordinate system, the north direction was the positive direction of Y axis, and the longitude and latitude of the center of each quadrat (transect and grid) were converted into corresponding coordinates to facilitate the analysis of egg density.

Based on the difference of topography and vegetation, we did not investigate the part of space between the two transects. The relevant areas are rocky slopes and hills, with sparse vegetation, and the density and coverage of Stipa caucasica are significantly lower than those of the transect and grid area (Additional file 1). The transect scheme has two very spaced cross sections, so we used the anisotropic semivariogram calculated along the transect direction, respectively.

**Fig. 2:**
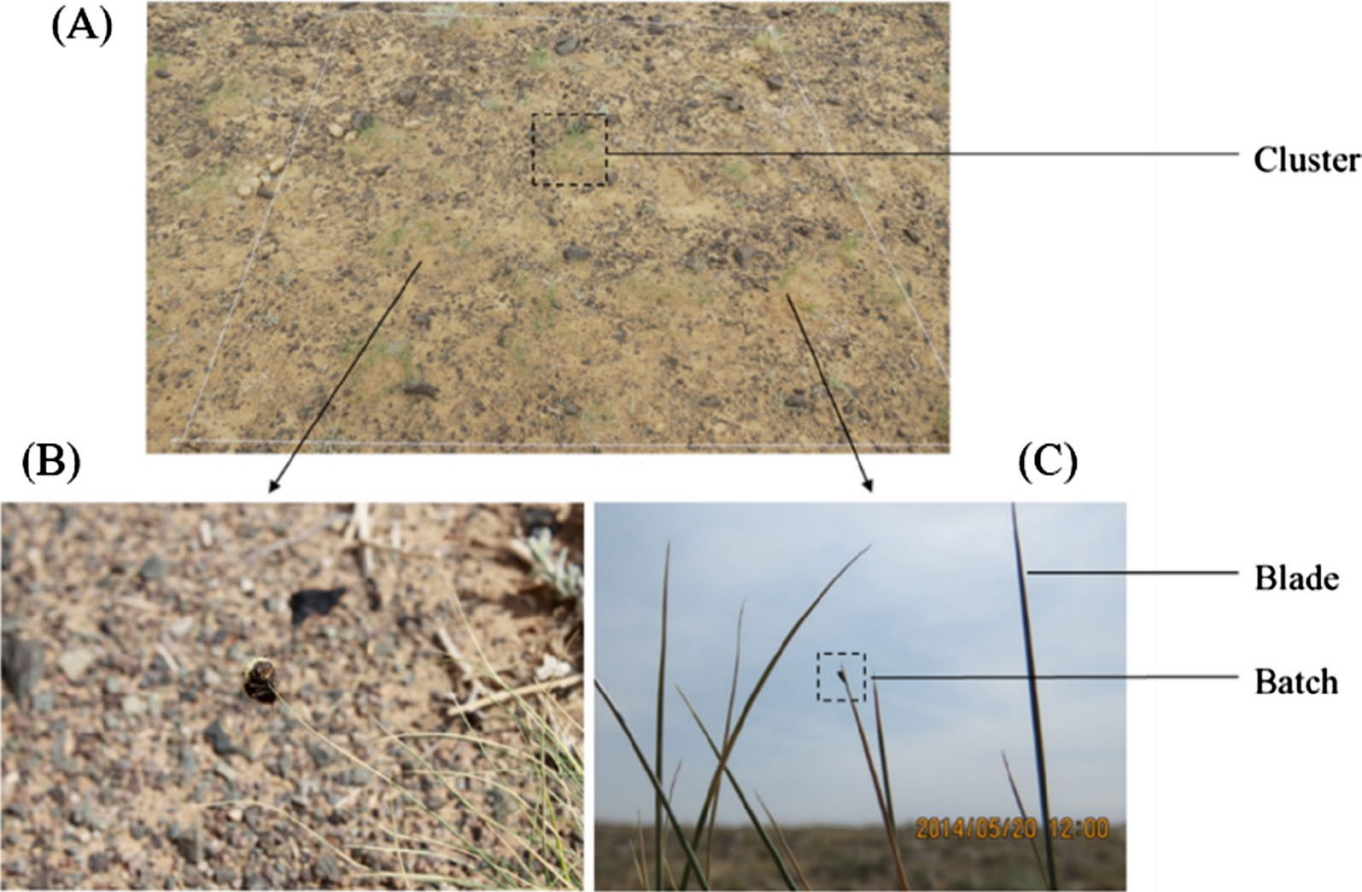
The *G. pecorum* eggs and *S. caucasica*. **A** The 2 m × 2 m investigative quadrat (This size is used to facilitate the display of vegetation). **B**, **C** The oviposition of *G. pecorum* in *S. caucasica*

For each quadrat, we first counted the number of *Stipa caucasica* in each quadrat and then carefully checked whether the *Gasterophilus pecorum* eggs were on them. If yes, then we continued to count the number of eggs. In 1 quadrat of each sample point, vegetation coverage, *S. caucasica* coverage, average height of *S. caucasica*, and oviposition on *S. caucasica* by *G. pecorum* were recorded. The oviposition count is quantified based on the number of *S. caucasica* clusters, blades and batches (the number of *G. pecorum* eggs deposited) (Fig. 2). The inspection method and quadrat size pattern described in the former section are described below.

### Statistical analysis

Semivariance is the mathematical expectation of incremental square of regionalized variables Z(*x*_*i*_) and Z(*x*_*i*+*h*_). Based on the theory of regionalized variables, semivariogram functions may be defined as,

$$\gamma (h) = \frac{1}{2n(h)}\sum {\left[ {{\text{Z}}(x_{i} ) - {\text{Z}}(x_{i + h} )} \right]^{2} }$$

where* γ*(*h*) is the function value of semivariance at the sample distance *h*, N(*h*) is the logarithm of sample pairs, (*x*_*i*_, *x*_*i*+*h*_), Z(*x*_*i*_) and Z(*x*_*i*+*h*_) are measured sample values at the sample point *x*_*i*_ and *x*_*i*+*h*_, and *h* is the distance of separation between any 2 sample points.

There are three key parameters of semivariance: nugget (C_0_), sill (C_0_ + C), and range (A). Nugget is the intercept at which lag distance equals zero, reflecting the degree of internal randomness of the regionalized variable. Sill is the value at which the semivariogram reaches equilibrium, revealing the rangeability of the variable. The difference (C) between the sill and nugget represents the proportion of the total variance. Spatial variability, which shows the relationship between this difference and the sill: (C/C_0_ + C), was used to reflect the degree of spatial dependence [16]. Range is the distance at which the semivariogram reaches a maximum, representing spatial dependence. The samples spatially autocorrelate within range.

The linear model, the spherical model, the Gaussian model, and the exponential model are common models of semivariance. Based on the principle of minimum error, the coefficient of determination (R^2^), range (A) and nugget (C_0_) were considered as selection factors [22].

Because eggs are deposited in batches of 10–15 [10], the proportion of ovigerous *S. caucasica* was selected to calculate and analyze the spatial distribution of *G. pecorum* eggs. The Mann–Whitney test was used to analyze the oviposition data in the two sampling methods. Semivariograms were generated using GS+ 6.0 and spatial distribution maps were created via Surfer 8. The relationship between ovigerous *S. caucasica* and environmental variables such as vegetation coverage, *S. caucasica* coverage, average height of *S. caucasica* and distance from water resources, were explored using Spearman’s rank correlation. Statistical analyses as well as plot graphics analyses were performed using SPSS version 20.0. Statistical significance was set at *p* ≤ 0.05.

## Results

### Quadrat size

To evaluate the optimal quadrat size, five different quadrat sizes were analyzed. Data obtained from them indicate that the frequency of eggs increased and the percentage of ovigerous *S. caucasica* decreased as the quadrat size increased. The means ($\overline{x }$) were relatively constant for quadrat lengths over 3 m. Exceeding a 4 m × 4 m area, the frequency of eggs was high and the coefficient of variance (CV) was low (Table 1). Therefore, considering the workload involved and the actual situation in the wild, the investigation was conducted using a sample area of 4 m × 4 m size.

**Table 1:** The comparison between different sampling area sizes for *G. pecorum* eggs in *S. caucasica*

Sampling area size	F	$\overline{x }$	μ	S	CV
1 m × 1 m	30	3.33	0.6	1.07	1.78
2 m × 2 m	60	1.94	1.4	1.90	1.36
3 m × 3 m	70	1.48	2.4	2.01	0.84
4 m × 4 m	80	1.35	3.9	2.47	0.63
5 m × 5 m	90	1.44	6.5	3.27	0.50

### Spatial distribution

**Fig. 3:**
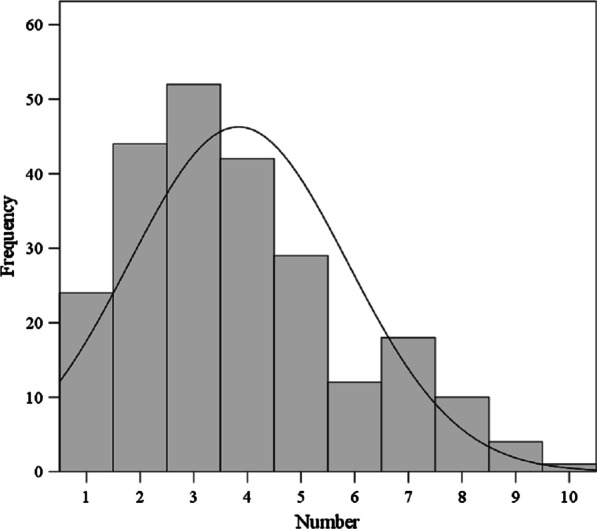
Frequency of *G. pecorum* eggs in batches

157 quadrats were sampled and 12,271 clusters of *S. caucasica* were examined. Eggs were detected in 63.1% of the quadrats (99/157) and 1.5% of the clusters of *S. caucasica* (187/12,271). Oviposition was observed in 187 clusters, on 236 blades of *S. caucasica* (Table 2). No significant difference in the percentage of ovigerous *S. caucasica* was observed between the two sampling methods (*t* = 0.107, *p* = 0.925)*.* Furthermore, 78.6% (147/187) of the *S. caucasica* clusters contained *G. pecorum* eggs in 1 blade, 16.6% (31/187) of the clusters contained *G. pecorum* eggs in 2 blades, and 4.8% (9/187) contained *G. pecorum* eggs in 3 blades. Eggs were deposited in batches of 1–10 (Fig. 3) and concentrated at the leaf apices of *S caucasica.* Over 95.0% of the ovipositioned eggs were present in batches of 1–8; the eggs were most frequently observed in batches of 3 (22.0%, 52/236) and 4 (19.5%, 46/236). The mean oviposition count was 3.8.

**Table 2:** Oviposition counts of *G. pecorum* in different sampling methods

Sampling method	Ovigerous quadrats /(%)	*Stipa caucasica* clusters	Ovigerous clusters /(%)	Ovigerous blades	Oviposition count (Mean ± SD)
Transect	40 (69.0%)	5068	81 (1.6%)	107	3.6 ± 1.5
Grid	59 (59.6%)	7203	106 (1.5%)	129	4.0 ± 1.7
Total	99 (63.1%)	12,271	187 (1.5%)	236	3.8 ± 1.6

**Fig. 4:**
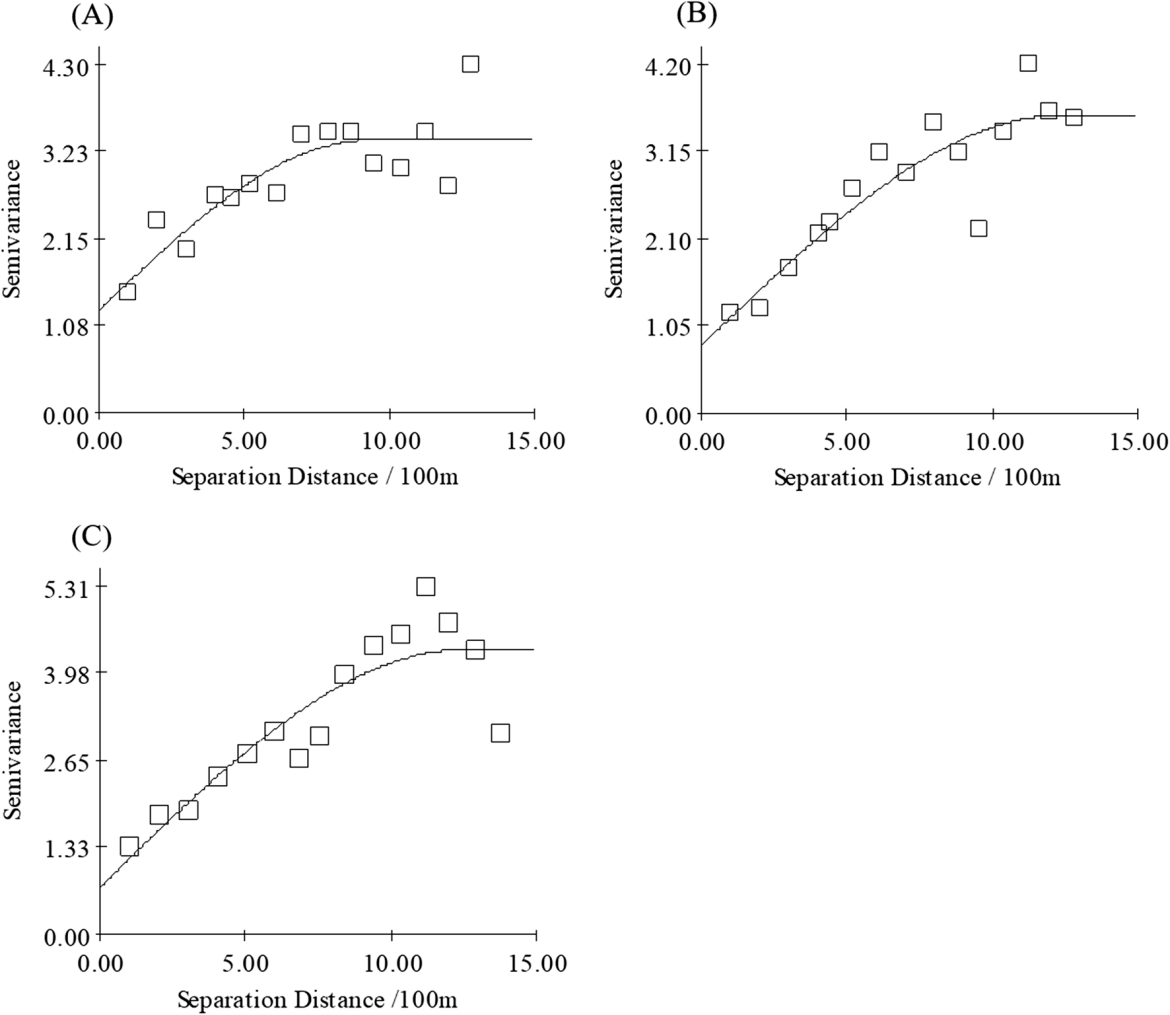
Variogram for *G. pecorum* eggs presence. Transect 1 (**A**), transect 2 (**B**) and grid method (**C**)

The spherical model provided the best fit (transect 1, R^2^ = 0.70; transect 2, R^2^ = 0.80; Grid, R^2^ = 0.89) for *G. pecorum* egg distributions in both sampling methods (Fig. 4). The spatial dependence (A) of the eggs was found to be 921 m (transect 1), 1233 m (transect 2) and 1097 m (grid), suggesting that the eggs were spatially independent at distances > 1233 m and > 1097 m in the transect method and grid method, respectively. The spatial variability (C/C_0_ + C) was 0.62 (transect 1), 0.77 (transect 2) and 0.57 (grid), indicating that structural variance, rather than nugget variance, was majorly responsible for the spatial dependence of the eggs. The different values of spatial variability depicted that the spatial autocorrelation of the eggs was more significant in the transect method.

**Fig. 5:**
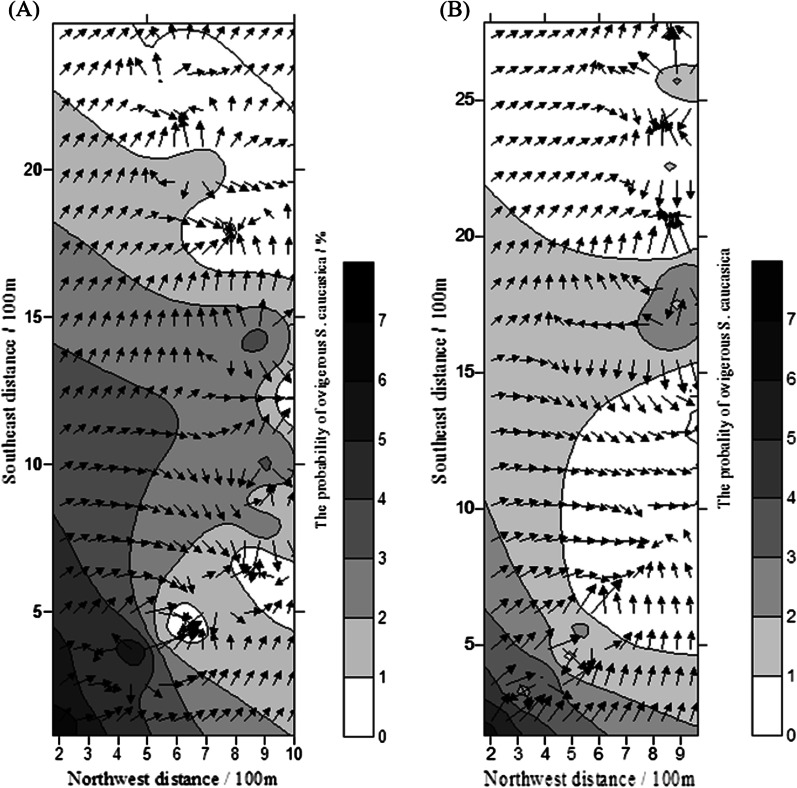
Overlay map of isoline and vector for the probability of ovigerous *S. caucasica*. Transect 1 (**A**) and transect 2 (**B**)

**Fig. 6:**
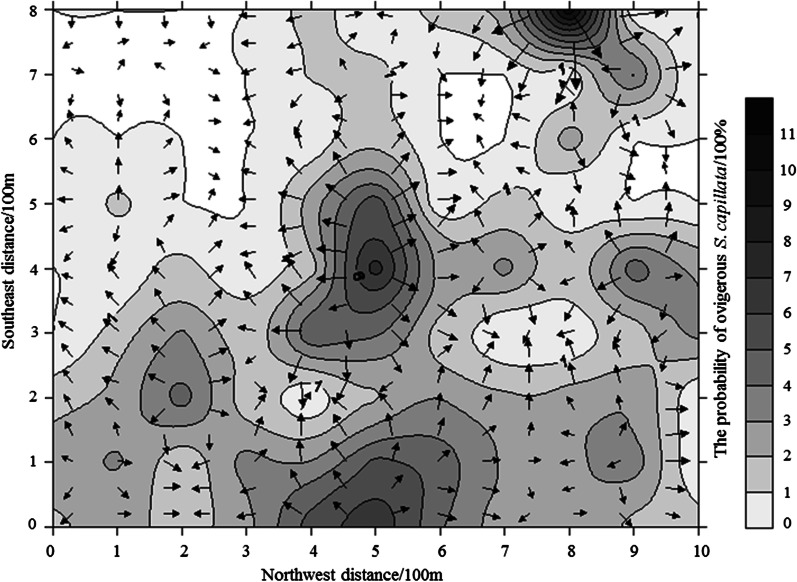
Overlay map of isoline and vector for the probability of ovigerous *S. caucasica* (grid)

The distribution map showed the range and magnitude of the spatial distribution of *G. pecorum* eggs in the two methods (Figs. 5 and 6). Generally, the eggs manifested a patchy and aggregated distribution pattern. More ovigerous *S. caucasica* were found near water resources; an inverse relationship emerged as the number of ovigerous *S. caucasica* declined gradually with increase in the distance from the water resource. In the transect method, the distribution of eggs was found to be more dispersed at low southwest distances, and after 2000 m from the water source, the probability of ovigerous *S. caucasica* was estimated to be almost zero. (Fig. 5).

**Fig. 7:**
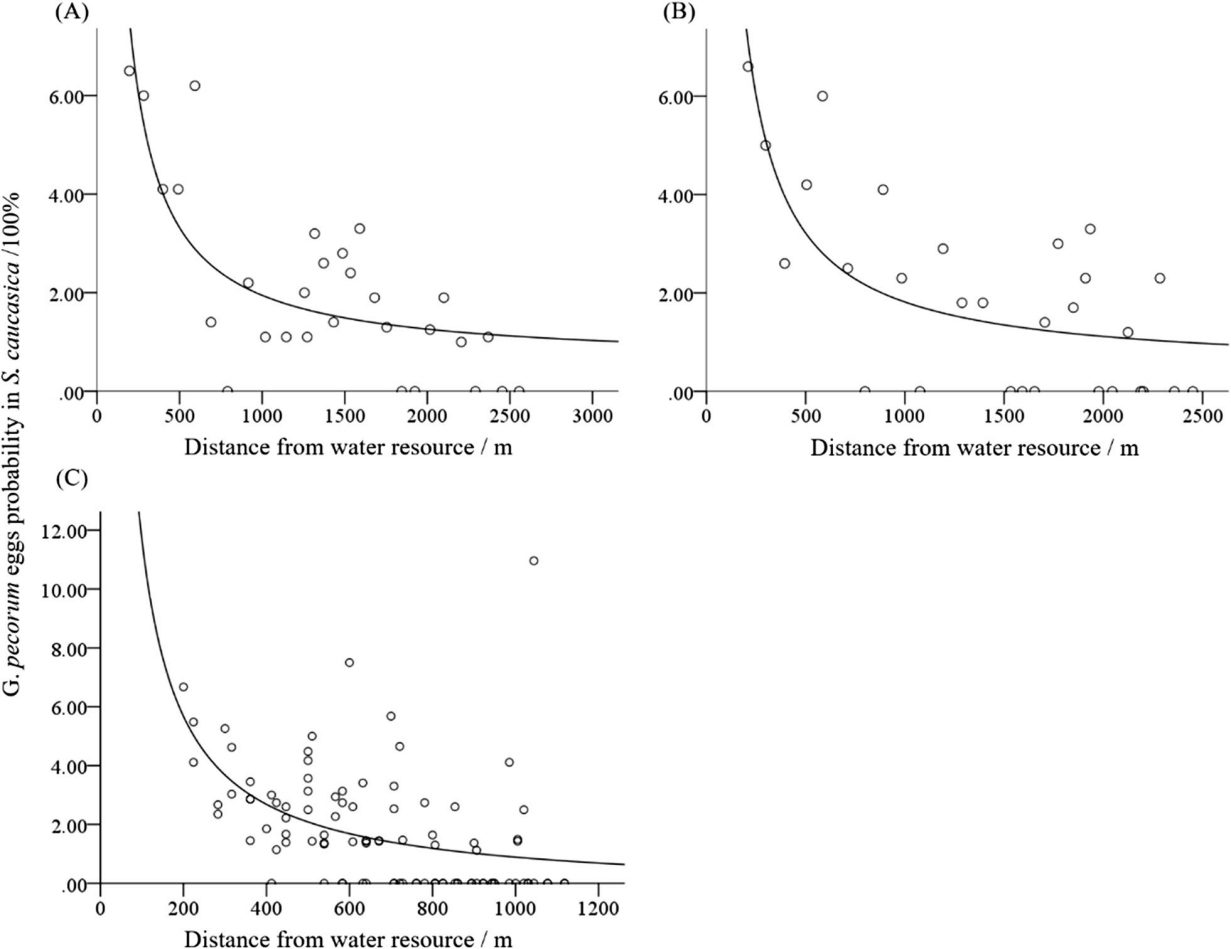
Correlation between the probability of ovigerous *S. caucasica* and distance from the water resource. Transect 1 (**A**), transect 2 (**B**) and grid method (**C**)

Correlation analysis revealed that the percentage of ovigerous *S. caucasica* was significantly negatively correlated (*r* = − 0.382, *p* = 0) with the distance from water resources (Table 3). The optimal model (*x*: distance from water resource, *y*: the probability of ovigerous *S. caucasica*) for egg distribution was the reciprocal model (Transect 1: $y=0.574+\frac{1372}{x}$, R^2^ = 60%, *p* < 0.001; Transect 2: $y=0.417+\frac{1399}{x}$, R^2^ = 52%, *p* < 0.001; Grid: $y=-0.309+\frac{1196}{x}$, R^2^ = 24%, *p* < 0.001). The density of *G. pecorum* eggs was high in the initial 500 m area around the drinking paths of equids (Fig. 7).

**Table 3:** Correlation between the probability of ovigerous *S. caucasica* and environmental factors

Factor	*r*	*p*
Vegetation coverage	0.115	0.151
*Stipa caucasica* coverage	0.018	0.820
Average height of *Stipa caucasica*	− 0.089	0.270
Distance from water resource	− 0.382	0.000

## Discussion

Kriging is a geostatistical technique used by ecologists to explicitly recognize hierarchical layering of patch structures in the spatial distribution pattern of species [23]. As a statistical tool, it must be combined with biological characteristics to reflect the relationship between arthropod distribution and ecological processes [24]. Based on the life history and traits of *G. pecorum*, we arranged sampling sites around a water resource and determined 4 m × 4 m to be the best sampling area size. This not only improved the accuracy of the spatial estimation, but also reduced the costs associated with labor, material, and time.

Carbajo et al. [16] have reported that the eggs of *Aedes aegypti* had no spatial dependence at 850 m intervals. This substantial nugget indicated that a study involving related species should utilize shorter intervals for trap placement. In the current study, 100 m intervals were selected for sampling the eggs of *G. pecorum,* which is a similar species belonging to the order *Diptera*. Spatial autocorrelation values of 62% (transect 1), 77% (transect 2) and 57.0% (grid) reflected the distribution pattern clearly.

Different sampling methods may affect the results of spatial distribution studies. Park and Obrycki reported that although temporal synchrony existed between lady beetles and aphids on a large scale, it was not obvious due to the magnitude of the scale [25]. In this study, similar semivariogram functions and spatial variability of both sampling methods exhibited a common distribution pattern. Larger distances may have increased the spatial autocorrelation of eggs, resulting in the spatial dependence estimated by the transect method being larger than that estimated by the grid method.

Our results, indicated oviposition counts between 1 and 10 with a mean of 4, were different from those of previous studies, which showed oviposition counts between 10 and 15 [10]. The fecundity of *G. pecorum* was reported to be in the range of 1300–2425 [1]. The lower oviposition count may have increased the spawning frequency, leading to a larger epidemic area. As a diversification strategy, it may increase the infection frequency of equids in sparsely vegetated areas more effectively. The spatial dependence (1250 m (transect) and 1097 m (grid)) of the *G. pecorum* eggs observed in this study, which was large when compared to previous studies’ estimations on the spatial distribution of insect eggs [15, 26], may be related to this unique behavior.

*G. pecorum* eggs displayed an aggregated distribution and were found mainly around water resources. The percentage of ovigerous *S. caucasica* was significantly correlated with the distance from the water resource. Furthermore, the spatial distribution of eggs was similar to that of equine feces [11]. These results confirmed the biological characteristics of *G. pecorum*, which does not pursue the host, but oviposits directly on grass [1].

## Conclusion

In this study, we focused on the oviposition of *G. pecorum* and explored its spatial distribution in the desert steppe. The smaller oviposition count observed in this habitat, which was low compared to that observed in Kazakhstan, may be due to the adaptation of this insect to the local environment. The peripheries of water resources were observed to be an important epidemic area for *G. pecorum*. Our findings suggested that the local government should develop control measures to decrease the density of eggs around this epidemic area. This may revitalize the efforts that are being taken to reintroduce Przewalski’s horses (Additional file 3).

## Supplementary Information

**Additional file 1: Figure S1.** Area between two transects.

**Additional file 2: Figure S2.** Drinking path of equids.

**Additional file 3: Figure S3.** Transect (path) and water source.

## Data Availability

All data generated or analyzed during this study are included in this published article and its additional information files.

## Abbreviations

KNR: Kalamaili Nature Reserve
HL: Hongliu (a water resource)
CV: Coefficient of variance
