# Supplementary figures and images for: Spatial distribution of Gasterophilus pecorum (Diptera) eggs in the desert steppe of the Kalamaili Nature Reserve (Xinjiang, China)
Source: BMC Ecol Evol. 2021 Sep 6;21:169. doi: 10.1186/s12862-021-01897-4 (PMC8422714; doi:10.1186/s12862-021-01897-4)

Additional file 1: Figure S1. Area between transects.


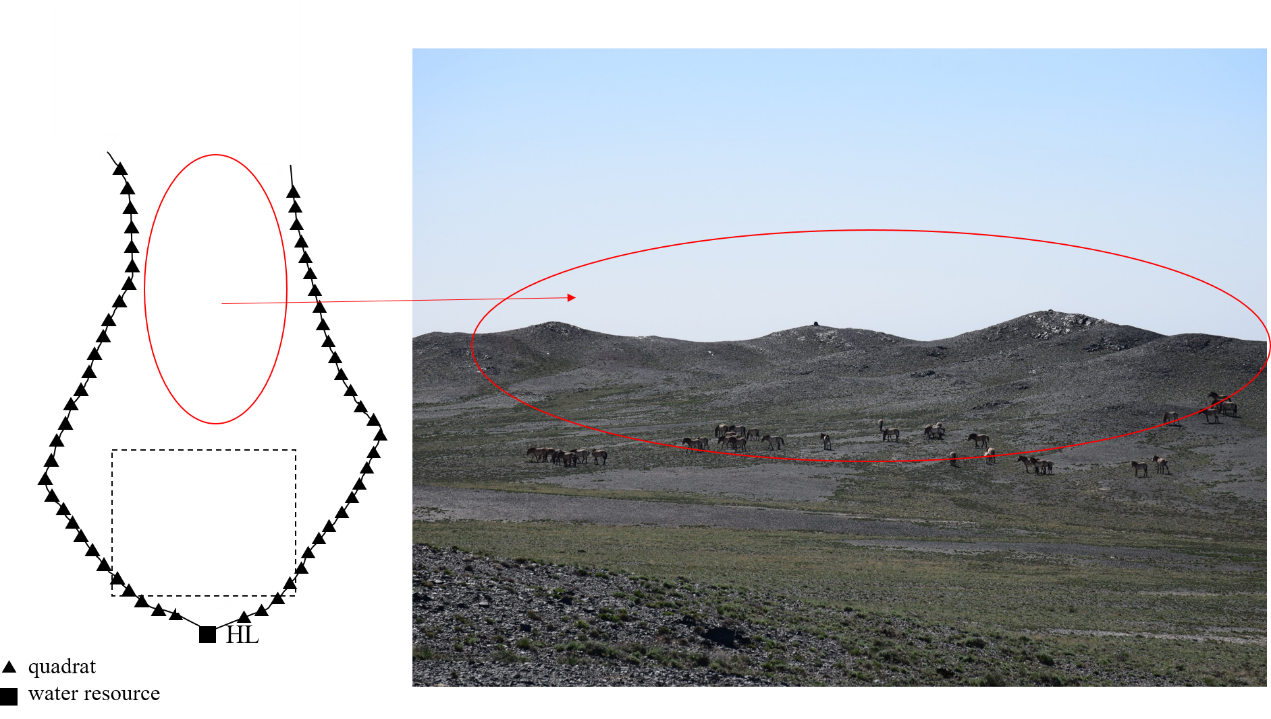

Supplement: Supplementary file 1 — Additional file 1: Figure S1. Area between two transects. [file 12862_2021_1897_MOESM1_ESM.docx]

Additional file 2: Figure S2. Drinking path of equids.


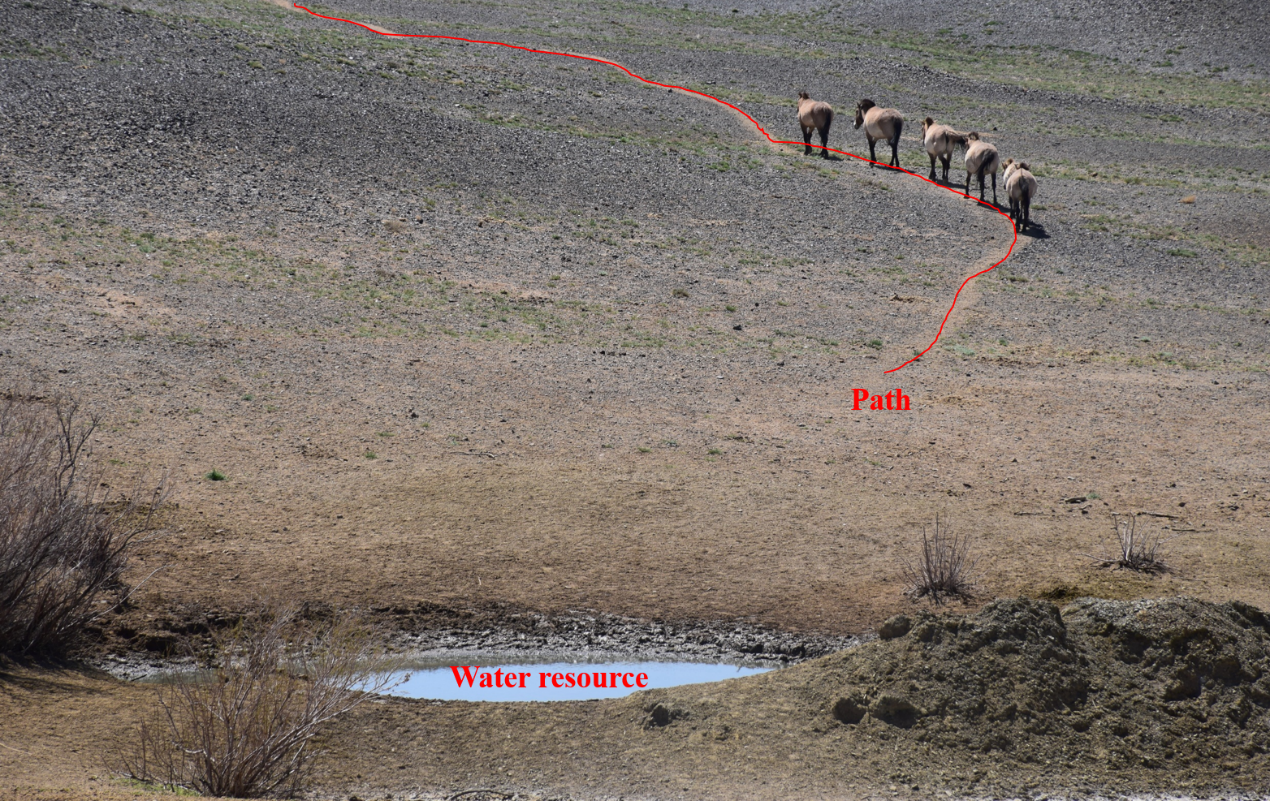

Supplement: Supplementary file 2 — Additional file 2: Figure S2. Drinking path of equids. [file 12862_2021_1897_MOESM2_ESM.docx]

Additional file 3: Figure S3. Transect (path) and water source.


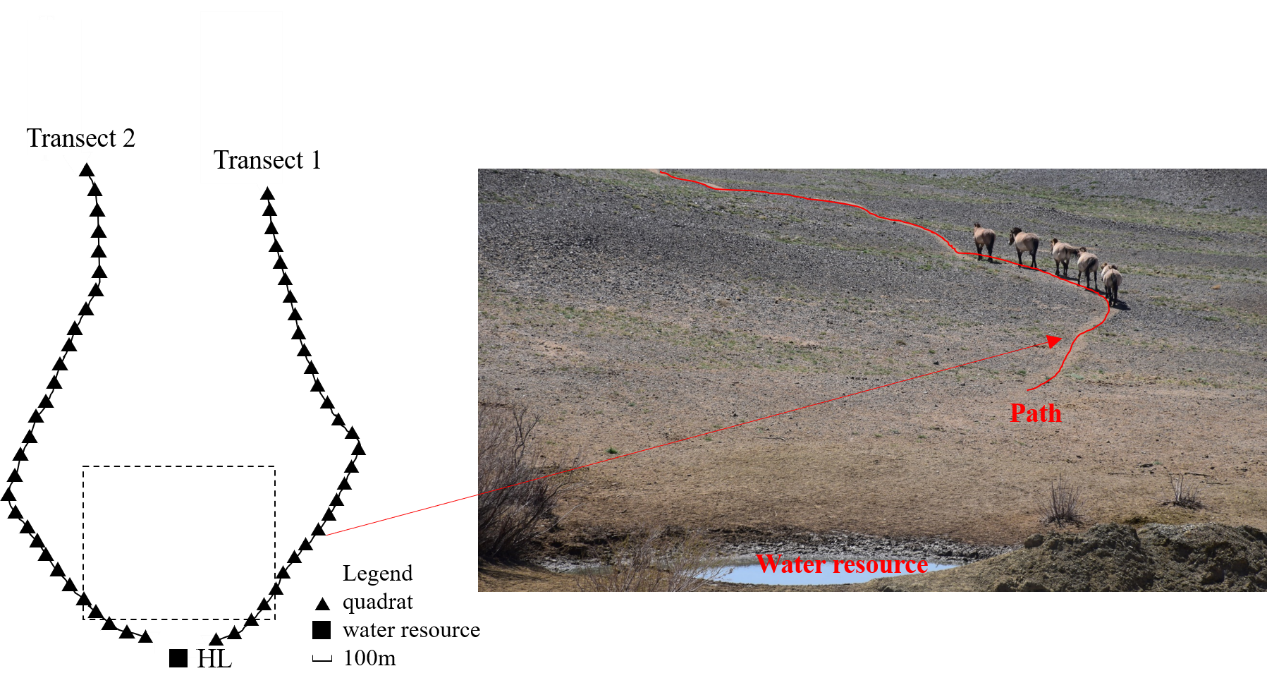

Supplement: Supplementary file 3 — Additional file 3: Figure S3. Transect (path) and water source. [file 12862_2021_1897_MOESM3_ESM.docx]
